# Structural brain alterations in primary open angle glaucoma: a 3T MRI study

**DOI:** 10.1038/srep18969

**Published:** 2016-01-08

**Authors:** Jieqiong Wang, Ting Li, Bernhard A. Sabel, Zhiqiang Chen, Hongwei Wen, Jianhong Li, Xiaobin Xie, Diya Yang, Weiwei Chen, Ningli Wang, Junfang Xian, Huiguang He

**Affiliations:** 1 State Key Laboratory of Management and Control for Complex Systems, Institute of Automation, Chinese Academy of Sciences, Beijing, China; 2 Department of Radiology, Beijing Tongren Hospital, Capital Medical University, China; 3 Department of Medical Psychology, Otto-von-Guericke University of Magdeburg, Medical Faculty, Institute of Medical Psychology, Magdeburg, Germany; 4 Research Center for Brain-inspired Intelligence, Institute of Automation, Chinese Academy of Sciences, Beijing, China; 5 Department of Ophthalmology, Beijing Tongren Hospital, Capital Medical University, China

## Abstract

Glaucoma is not only an eye disease but is also associated with degeneration of brain structures. We now investigated the pattern of visual and non-visual brain structural changes in 25 primary open angle glaucoma (POAG) patients and 25 age-gender-matched normal controls using T1-weighted imaging. MRI images were subjected to volume-based analysis (VBA) and surface-based analysis (SBA) in the whole brain as well as ROI-based analysis of the lateral geniculate nucleus (LGN), visual cortex (V1/2), amygdala and hippocampus. While VBA showed no significant differences in the gray matter volumes of patients, SBA revealed significantly reduced cortical thickness in the right frontal pole and ROI-based analysis volume shrinkage in LGN bilaterally, right V1 and left amygdala. Structural abnormalities were correlated with clinical parameters in a subset of the patients revealing that the left LGN volume was negatively correlated with bilateral cup-to-disk ratio (CDR), the right LGN volume was positively correlated with the mean deviation of the right visual hemifield, and the right V1 cortical thickness was negatively correlated with the right CDR in glaucoma. These results demonstrate that POAG affects both vision-related structures and non-visual cortical regions. Moreover, alterations of the brain visual structures reflect the clinical severity of glaucoma.

Glaucoma is the second leading cause of blindness and has been the topic of intense study to uncover the underlying mechanisms of the disease. By comparing retinal nerve fiber layer (RNFL)^1^,^2^,^3^ and macular thickness^1^,^4^,^5^ between normal controls and glaucoma patients, it was shown that the fundus is altered in glaucoma. Gupta *et al*.^6^ were the first to demonstrate that glaucoma is associated not only with optic nerve degeneration but also with structural changes of the lateral geniculate nucleus (LGN) of the thalamus and visual cortex. This suggests that beyond ocular and optic nerve structures, glaucoma also affects central structures of the visual pathway in the brain. These central visual pathway alterations in glaucoma along the optic radiation have been confirmed by others^7^,^8^,^9^, and altered functional organization of V1 was shown using blood oxygenation level dependent imaging in glaucoma patients as well^10^.

In fact, glaucoma has been thought of as a neurodegenerative disease^11^ for years. Previous studies showed that intracranial pressure (ICP) changes have an impact not only on the optic nerve but also on other brain regions^12^,^13^. Especially, watershed areas of brain circulation are particularly vulnerable to ICP changes^14^. These are regions where the different brain arterial regions overlap, i.e. the anterior, middle and occipital artery branches. Whereas the confluent regions of the middle/occipital watershed areas may explain visual system loss, ICP changes in glaucoma may also impact the non-visual anterior/middle cerebral artery territory. These studies stress the need of studying the whole brain of glaucoma patients, not just the central visual structures.

Morphological brain changes were observed even outside the visual pathway throughout the whole brain as shown by different methods^3^,^15^,^16^,^17^. For example, Williams *et al*. measured the volumes of 93 structures in the brain and observed widespread structural brain changes even in non-visual structures such as the bilateral inferior occipital gyri, right inferior temporal gyrus and other structures as well^16^. Furthermore, Yu *et al*.^17^ observed reduced cortical thickness in calcarine sulci, the left middle temporal gyrus and fusiform gyrus.

However, the aforementioned studies of the whole brain structures just focused on structural changes using a single morphological marker. Moreover, some of these studies suffered from the methodological flaw of using relatively liberal corrections for multiple comparisons during statistical analyses or even no correction at all. This raises the question how widespread brain morphological alterations really are.

To overcome these limitations, we now investigated brain structural changes in glaucoma patients using three independent analysis tools: volume-based analysis (VBA), surface-based analysis (SBA) and ROI-based analysis. In the statistical process, the most conservative family-wise error (FWE) correction was applied to reduce the likelihood of type 1 errors using multiple comparison statistics. Finally, we correlated the morphological markers with the clinical measures.

## Methods

### Subjects

The study was conducted according to the Declaration of Helsinki and was approved by the Committee at Beijing Tongren Hospital. Written informed consent was obtained from all the subjects which comprised 25 primary open angle glaucoma (POAG) patients (age: 44.6 ± 13.0 years, male/female: 11/14) and 25 age-sex-matched normal controls (age: 36.8 ± 11.6 years, male/female: 13/12).

All the normal controls underwent a routine clinical examination, which included optometric examination, intraocular pressure examination, inquiry of personal disease history and family disease history. This was done to assure that they had no glaucoma or other visual diseases. The diagnoses of POAG in patients were based on a thorough history and ophthalmologic examinations, including optic intraocular pressure, gonioscopy, fundoscopy and standard automated perimetry. The diagnosis criteria included high intraocular pressure, open anterior chamber angle, typical vision loss, glaucomatous abnormalities of the optic nerve head and optic disk. The glaucoma diagnosis was made by three independent glaucoma clinicians in a masked (blinded) manner.

### Acquisition of clinical measures and MRI data

The patients underwent 30-2 Swedish Interactive Testing Algorithm (SITA) fast visual field tests with standard automated perimetry (Humphrey Field Analyzer; Zeiss Meditec AG, Jena, Germany) to measure visual field loss (mean deviation: MD). The iVue 100 (Optovue Inc, Fremont, California, USA) was used to measure CDR and RNFL thickness. Because all patients were recruited from the outpatient service between 2009 and 2012, which was a long time before the current retrospective research was carried out, some clinical data of ophthalmologic examinations were missing when assembling the medical records for our study so that about half of our patients had incomplete data sets and could not be included in the correlation analysis. All the available clinical data of POAG patients (n = 14) are listed in Table 1.

Because RNFL thickness, CDR and the visual field MD are the three most commonly used clinical parameters to diagnose glaucoma, these parameters were correlated with the abnormal brain morphological measures.

Brain MRI scans were made in a GE 3T scanner to acquire T1-weighted structural MRI images with the following scanner parameters: TR/TE = 8.9/3.5ms, slice thickness = 1 mm, flip angle = 13^o^, matrix = 256 × 256, FOV = 24 × 24 cm^2^.

### Data Analysis

**Figure 1:**
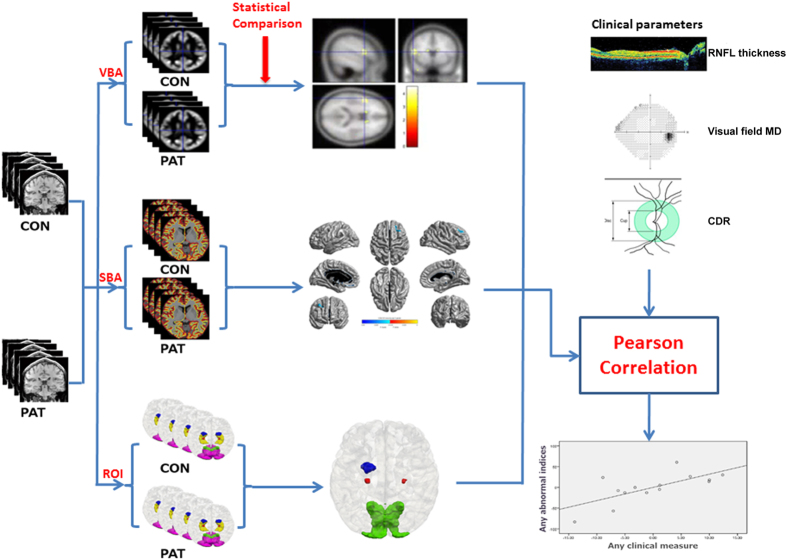
The pipeline of the structural brain abnormality in glaucoma patients. CON: normal controls, PAT: glaucoma patients, VBA: voxel-based analysis, SBA: surface-based analysis, ROI: ROI-based analysis, RNFL: retinal nerve fiber layer, CDR: cup-to-disk ratio, MD: mean deviation.

We investigated structural abnormalities in glaucoma with three independent measures: VBA, SBA and ROI-based analysis. For VBA, gray matter volume was defined by voxel-based morphometry (VBM). SBA assessed the vertex-based cortical thickness and for the ROI-based analysis, we studied the volumes of five selected brain structures in each hemisphere (LGN, V1, V2, amygdala and hippocampus). Finally, in glaucoma patients we correlated brain morphological measures with retinal measures and vision loss as quantified by mean deviation obtained by standard automated perimetry (Fig. 1).

#### Volume-based analysis

All T1-weighted images were analyzed by VBM with DARTEL in Statistical Parametric Mapping software (SPM8, http://www.fil.ion.ucl.ac.uk/spm/).The image processing included GM/WM/CSF segmentation of all T1 weighted images^18^, GM/WM template generation with DARTEL method^19^, spatial normalization, volume modulation, and spatial smoothing with a 4mm Gaussian kernel. Subsequently, the abnormality of GM volume between both groups was analyzed by two sample *t*-test (age and gender as covariates) and corrected by FWE of 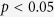.

#### Surface-based analysis

We compared the vertex-based cortical thickness to implement the surface-based analysis. The cortical thickness was calculated by FreeSurfer software version 5.1 (http://surfer.nmr.mgh.harvard.edu/). The image process included non-uniform intensity correction, skull-strip of the brain^20^, construction of the white surface and the pial surface^21^ and cortical thickness calculation^22^. Finally, the vertex-based thickness of each subject was normalized to a template provided by FreeSurfer. We subsequently conducted two sample *t*-tests to assess the thickness differences between two groups with age and gender as covariates (FWE corrected, 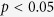).

#### ROI-based analysis

**Figure 2:**
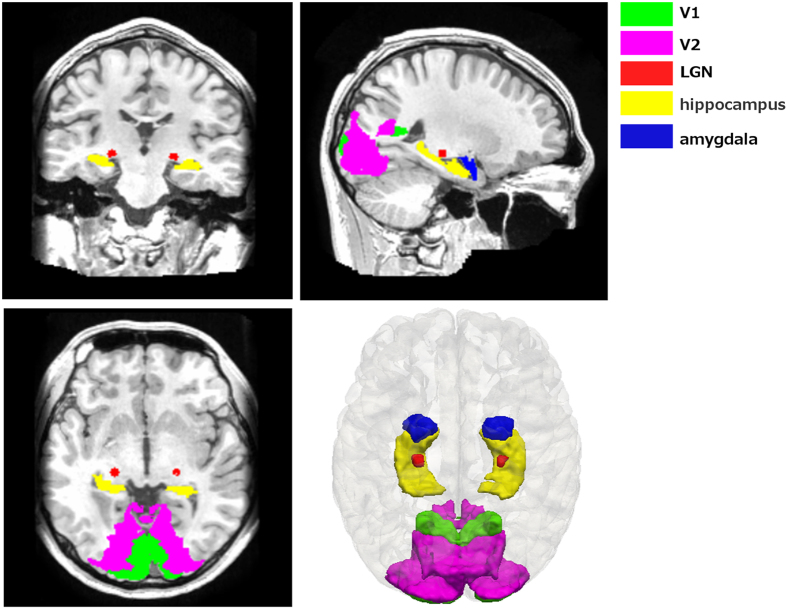
The visualization of structures in the ROI analysis.

Because glaucoma is a visual disease, ROI-based analysis focused on three vision-related structures: LGN, V1, and V2. Moreover, as glaucoma patients were reported to have emotional^23^,^24^ and memory impairments^25^, we also investigated possible volume alterations of two emotion/memory-related structures, i.e. amygdala and hippocampus (Fig. 2). These structures (with the exception of LGN) were all segmented by FreeSurfer. The delineation of V1 and V2 was labeled based on an atlas of the Martinos Center for Biomedical Imaging (http://www.nmr.mgh.harvard.edu/)^26^. The segmentation of amygdala and hippocampus was conducted based on a probabilistic atlas^27^ and the LGN delineation by an automatic LGN segmentation approach^28^. After the ROI segmentation, two-sample *t*- tests were conducted to compare the volume of each ROI between the two groups controlling for age and gender (Note that in a prior publication we have reported the LGN structural results to validate the feasibility of an automatic quantification method for structural analysis^28^).

#### Correlation with clinical measures

In order to further investigate the relationship between the morphological abnormalities and the visual functions, morphological abnormalities was correlated with RNFL thickness, CDR and the visual field MD using SPSS (release 17.0) by taking into account the effect of age, gender and intracerebral volume (ICV).

## Results

### Volume- and surface-based analyses

**Figure 3:**
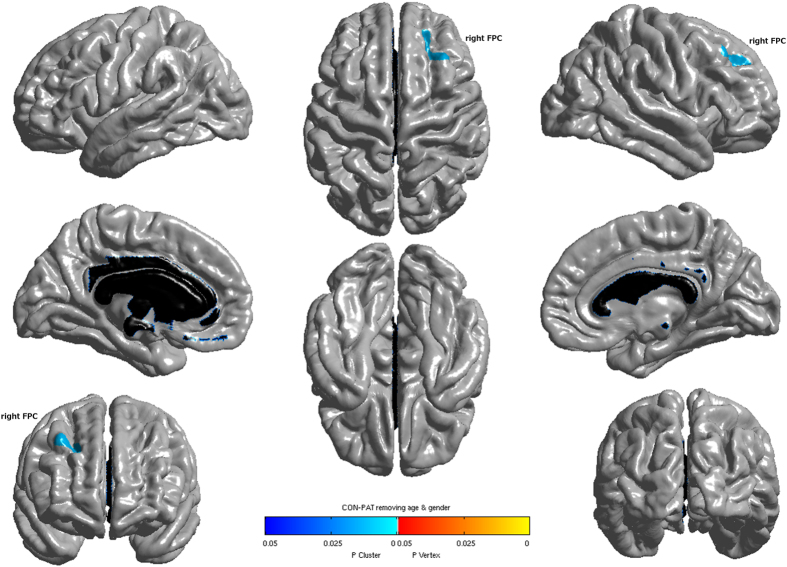
Cortical regions with significantly reduced cortical thickness in glaucoma patients when compared with normal controls (P<0.05, FWE corrected). FPC: frontal pole cortex.

Glaucoma patients showed no significant GM volume alterations in any region compared to normal controls. However, surface-based analysis revealed significantly reduced thickness of the right frontal pole cortex (FPC) in glaucoma patients (Fig. 3 and Table 2).

### ROI-based analysis

When compared to normal controls, glaucoma patients had significant volume shrinkages in the LGN bilaterally (left: 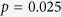, right: 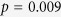) and in the right V1 (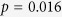) with no difference in V2 (Table 3). In order to investigate the possible reason of the right V1 atrophy, we compared its thickness and surface area. While the right V1 thickness was significantly reduced in glaucoma (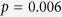), there was no significant change in the surface area (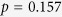). Therefore, it is the thinner V1 thickness of the glaucoma patients that may explain the overall atrophy of V1 volume. After comparing the amygdala and hippocampus volumes between the two groups, we found that only the left amygdala volume of glaucoma patients significantly decreased (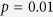), not the hippocampus (left: 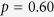, right: 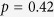)(Table 3).

### Relationship of brain morphology and clinical measures

**Figure 4:**
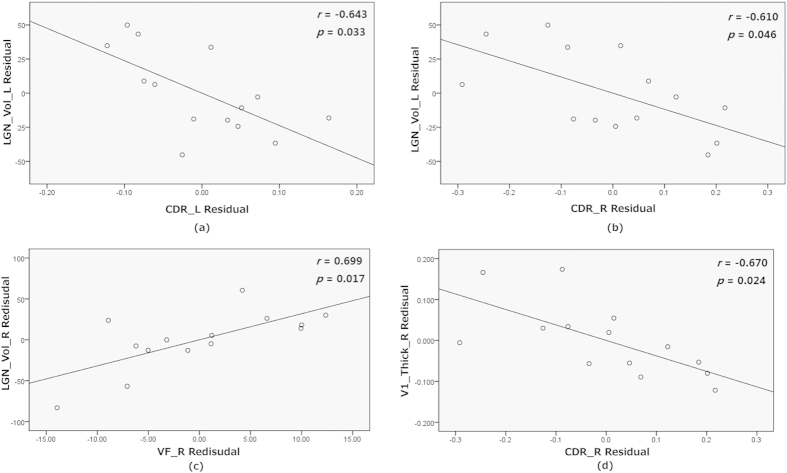
The partial correlation between the corresponding value of abnormal regions and clinical parameters with age, gender and intracerebral volume as covariates. LGN_Vol_L/R: left/right LGN volume, V1_Thick_R: right V1 cortical thickness, CDR_L/R: left/right cup to disk ratio, VF_R: right visual field mean deviation. M Residue: the difference between M (the observed value) and the result (the theoretical value) of linear regression of M with age, gender and intracerebral volume.

Correlation coefficients were calculated between brain morphological markers of glaucoma patients (right FPC thickness, bilateral LGN volumes, right V1 thickness, and left amygdala volume) and retinal morphological marker (RNFL thickness and CDR) or the visual field MD (see Table 4). The left LGN volume was negatively correlated with bilateral CDR (left: 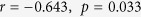, right: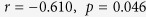) (Fig. 4(a,b)), i.e. a greater CDR was associated with greater LGN degeneration. The right LGN volume was positively correlated with the right visual field MD, i.e. small right LGN volume was associated with smaller visual field MD (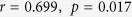) (Fig. 4 (c)). Furthermore, we observed a tendency of a positive correlation between the right LGN volume and the left visual field MD as well (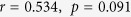) and noted that the right V1 cortical thickness was negatively correlated with right CDR (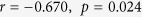) (Fig. 4(d)). Thus, similar to the LGN, degeneration of the primary visual cortex was associated with a greater CDR. Both the right FPC thickness and the left amygdala volume were not correlated with retinal morphology nor any other visual field measures.

## Discussion

By applying VBA, SBA and ROI-based analysis, we were able to confirm brain structural changes in the primary visual system in glaucoma patients^6^,^7^,^8^,^9^,^10^ which are correlated with clinical measures (RNFL thickness, CDR and visual field MD). In addition, there were independent morphological signs of degeneration in non-visual structures.

### Volume shrinkage of vision-related structures

In central visual structures of glaucoma patients we noted abnormalities in the primary visual relay center (LGN) and the primary visual cortex (V1). The LGN is an important visual relay center transferring visual information from retina to primary visual cortex^29^ that processes information about static and moving objects^30^,^31^. The volume atrophy we observed in these two structures is in agreement with prior observations of the degeneration of the central visual pathway in glaucoma^6^,^7^,^8^,^9^,^10^. The correlation of this degeneration with vision loss is not surprising but confirms, for the first time, that central degeneration contributes to the vision loss. Clearly, glaucoma is not a disease limited to the eye and optic nerve, but it is also a brain disease.

### Reduced FPC thickness

Surface-based analysis of whole brain morphology revealed that only the right FPC was found to be thinner in glaucoma patients compared to normal controls. This is compatible with other structural imaging studies showing protracted change in the FPC gray matter volume^32^,^33^ and thickness^34^ with normal human aging. Because our normal controls were age-matched, our finding shows that the FPC changes are greater in glaucoma than in normal aging.

FPC is also altered in other diseases, such as schizophrenia^35^ and autism spectrum disorders^36^. Thus, glaucoma, which is mostly a disease of the elderly, is associated with brain changes that resemble normal aging effects of brain degeneration but they are more severe. This is why glaucoma has also been referred to as an “eye dementia”^37^ . FPC thinning in glaucoma may be a sign of the susceptibility of the FPC, an important brain structure involved in many aspects of cognition^38^,^39^. Therefore, a thinner FPC may explain why some glaucoma patients have cognition impairments, especially if the glaucoma patients also have concomitant Alzheimer’s disease (AD)^40^. In addition, although FPC is found to be related to human cognition, the fundamental function and mechanism of FPC remains unclear^38^. Whether and to what extent FPC thinning contributes to the emergence of vision loss remains to be studied in greater detail. But recent studies^41^,^42^ observed that occipito-frontal functional connectivities are disturbed when the optic nerve is damaged^41^ and the functional connectivities improve when vision recovers^42^. These observations indicate that the FPC and impaired vision in glaucoma are related, possibly because of impaired attention, eye movement control (of the frontal eye fields) or cognitive processing. And there are other changes in non-visual structures, which may or may not contribute to vision loss.

### Alterations of emotion/memory-related structures

Another main finding of our study is that glaucoma patients suffer from abnormalities in the amygdala as well, which adds an emotional component and suggests that glaucoma is not just a pure “vision disorder” but a more complex brain syndrome. The amygdala plays a role in the control of emotion^43^,^44^,^45^, especially in fear and anxiety control. In fact, emotional changes in glaucoma patients have been reported before^23^,^24^, but we are the first to report volume atrophy in the amygdala in glaucoma which may explain why patients tend to be more anxious and easily experience anger^23^,^24^. Please note that the abnormality in the amygdala found in the study just focuses on the volumetric differences only; we have not studied functional changes of the amygdala in our glaucoma patients which should be investigated in future studies.

There is a close connection of the amygdala with the hippocampus, both of which are part of the Papez circuit that regulates emotion. The hippocampus is a structure controlling memory formation and its main function is to consolidate information from short-term memory to long-term memory. Indeed, memory impairments were reported in some glaucoma patients^25^. This is why we also analyzed the hippocampus to more fully grasp the neurobiological basis of psychological changes beyond visual perception in glaucoma. However, our morphometric analysis did not reveal any significant alterations of the hippocampus when glaucoma patients were compared to normal controls. As the previous study^25^ pointed out that memory impairment was found in only approximately 20% of the glaucoma patients, our sample size may be too small to reveal any hippocampus volume differences. To obtain a better understanding of the role of the hippocampus, in future studies it would be worthwhile to study brain structures in glaucoma patients with versus without memory problems.

### Correlations with clinical measures

Visual field MD and CDR are important clinical parameters in glaucoma diagnosis. The visual field MD is a functional sign of pathology; the smaller the visual field MD, the greater the extent of damage in glaucoma and the greater is the CDR. Both the right FPC thickness and the left amygdala did not correlate with standard clinical measures of disease severity. This indicates that the non-visual structural changes may occur independently of the atrophy of the optic nerves and other brain structures in glaucoma. The positive correlation between the right visual field MD and the right LGN volume is consistent with our expectations that they are related with each other in glaucoma. Furthermore, the negative correlation between bilateral CDR and left LGN volume as well as between right CDR and right V1 thickness is also a sign that a higher CDR is associated with degeneration in LGN and V1. The correlation results suggest that abnormalities in central visual structures reflect the glaucoma severity and demonstrate that the abnormal indices found by us may be used as the biomarker of the glaucoma.

### Lateralization of brain structural changes

It is unclear why in our study significant structural alterations were found only in one brain hemisphere: a thinner right FPC, right V1 and left amygdala. This is rather surprising since the damage to the optic nerve should affect both hemispheres equally. Because an asymmetric loss of the visual field can not explain the laterality (temporal and nasal retina were equally affected), we rather suspect that the lateralization of brain structural changes shows that the peripheral and central visual degeneration may not be controlled by the exact same factors; in other words, central degeneration does not simply mirror the peripheral damage in a one-to-one (retinotopic) manner. One possible independent component might be brain lateralization^46^ or eye dominance, but this needs further study.

### Comparison of VBA and SBA

Our study confirms that glaucoma patients suffer from brain structural abnormalities as demonstrated by volume-based and surface-based analyses. When a FWE correction was applied during statistical analysis, significant structural brain alterations were only seen when the surface-based analysis was used. This demonstrates that surface-based morphometry is the more sensitive method to reveal brain alterations than volume-based analysis, which is consistent with previous studies^34^,^47^,^48^,^49^.

### Discrepancies with previous studies

A volume-based analysis did not reveal any significant alterations between glaucoma patients and normal controls. This result differs from studies by Li *et al*.^15^ and Chen *et al*.^3^, who reported significant brain morphological changes in many cortical regions such as lingual gyrus, calcimine gyrus, etc. In a surface-based analysis, we found reduced cortical thickness only in the right FPC, which is different with Yu’s work^17^. There may be two reasons for the discrepancies of our results with those of others. Firstly, the three studies used different multiple comparison correction methods with only small volume correction (SVC)^3^ or no corrections at all^15^,^17^. In contrast, we have used FWE correction, a much stricter criterion than applying SVC or no correction at all, giving us more reliable results. Secondly, the kind of pathologies and the number of subjects are different between studies. Whereas in Li’s work the structural alterations were not found in the early stage but only in the late stage of POAG, this suggests that different stages of POAG patients used in the study may have impacted the results. In the present study we did not differentiate the POAG stages as both early and late stage POAG patients were included in our study.

### Limitations and future work

There are some limitations of our study. Firstly, the sample size of the cross-sectional design is relatively small, because only a smaller subsample of 14 out of 25 POAG patients had complete clinical records. Secondly, although the ages of two groups match statistically (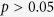), the difference of the mean age between these two groups was eight years. This might limit somewhat the interpretation of our findings. Yet, the correlation analyses of structural parameters and clinical parameters in our patients validate the clinical relevance of our study despite of this limitation.

When considering all available evidence^6^,^7^,^8^,^9^,^10^ it seems clear that glaucoma is highly correlated with changes in visual/non-visual cortical structures and optic nerve degeneration. But it is unknown whether and to what extent these three factors are dependent, i.e. if they have a common causal, pathological basis. Therefore, future studies should address this issue. Moreover, the visual information is probably processed by the primate visual brain with a network model^50^. Thus, the loss of visual functions may, in fact, relate to alterations of brain network patterns, which is also worth investigating in greater detail.

## Conclusions

In summary, brain structural alterations not only affect vision-related structures but also other non-visual cortical regions. These alterations are consistent with the syndromes of the glaucoma patients, e.g. dysfunction in visual perception. The correlations between the morphological alterations and clinical parameters suggest that the brain structural alterations reflect the glaucoma severity and may serve as a possible biomarker of glaucoma progression.

## Additional Information

**How to cite this article**: Wang, J. *et al*. Structural brain alterations in primary open angle glaucoma: a 3T MRI study. *Sci. Rep*. **6**, 18969; doi: 10.1038/srep18969 (2016).

## Figures and Tables

**Table 1:** The details of the clinical characteristics of the glaucoma patients.

ID	Sex	Age	RNFL thickness (μm)		CDR		Visual field MD (dB)	
			R	L	R	L	R	L
**1**	**F**	**17**	**102.22**	**106.49**	**0.60**	**0.57**	**−2.67**	**−3.51**
**2**	**F**	**47**	**73.71**	**76.08**	**0.66**	**0.61**	**−10.57**	**−12.14**
3	M	56	—	—	—	—	−6.55	−28.44
**4**	**M**	**59**	**78.85**	**77.26**	**0.90**	**0.87**	**−4.49**	**−4.27**
**5**	**M**	**52**	**84.35**	**77.38**	**0.56**	**0.71**	**−4.29**	**−6.59**
**6**	**M**	**60**	**148.87**	**77.38**	**0.91**	**0.74**	**−25.21**	**−3.85**
7	F	56	—	—	—	—	−6.77	−20.37
8	F	23	—	—	—	—	−10.30	−13.20
**9**	**M**	**68**	**60.01**	**61.45**	**0.90**	**0.91**	**−20.00**	**−28.81**
**10**	**M**	**18**	**64.50**	**104.26**	**0.73**	**0.43**	**−11.84**	**0.28**
11	F	46	—	—	—	—	—	—
12	M	28	—	—	—	—	−16.58	−26.90
**13**	**F**	**48**	**71.66**	**72.64**	**0.92**	**0.59**	**−14.99**	**−11.52**
14	F	58	—	65.34	1.00	0.89	—	−30.16
**15**	**M**	**29**	**64.59**	**68.74**	**0.94**	**0.70**	**−33.12**	**−14.51**
16	F	47	—	—	—	—	—	—
**17**	**F**	**40**	**80.58**	**74.97**	**0.32**	**0.50**	**−1.09**	**−1.11**
**18**	**M**	**55**	**102.06**	**104.29**	**0.98**	**0.87**	**−7.95**	**−3.58**
19	F	22	—	—	—	—	−23.50	−20.70
20	F	21	—	—	—	—	—	—
**21**	**M**	**75**	**64.16**	**96.69**	**0.97**	**0.95**	**−28.51**	**−6.88**
**22**	**F**	**41**	**103.16**	**90.03**	**0.07**	**0.42**	**−4.95**	**−6.47**
23	M	59	64.38	76.90	0.84	0.87	—	—
24	F	47	—	—	—	—	−16.18	−26.86
**25**	**F**	**44**	**77.04**	**72.05**	**0.69**	**0.51**	**−4.15**	**−5.26**
Mean(std)*			83.98 (17.25)	82.84 (12.51)	0.73 (0.21)	0.67 (0.15)	12.42 (8.54)	−7.73 (5.15)
Range*			[60.01, 148.87]	[61.45, 106.49]	[0.07, 0.98]	[0.42, 0.95]	[−33.12, −1.09]	[−28.81, 0.28]

M: male, F: female, RNFL: retinal nerve fiber layer, CDR: cup-to-disk ratio, MD: mean deviation. **Bold**: patients with all three parameters. *statistics were based on those data in **bold**.

**Table 2:** Cortical regions with significantly reduced cortical thickness in glaucoma compared to normal controls (p<0.05, FWE corrected).

Anatomical location	cluster size	p-value	x	y	z	t-value
Right frontal pole cortex	1059	0.01	22	36	36	3.89
			34	21	46	3.82

*x, y, z*: the MNI coordinates of the peaks in the cluster.

**Table 3:** Volume comparisons of five structures between normal controls and glaucoma patients in ROI analysis.

	Structures	Hemisphere	Controls	Patients	*p-value*
Vision-related structures	LGN	L	144.08 (32.63)	124.00 (28.48)	0.025^*^
		R	116.80 (29.83)	90.84 (37.47)	0.009^*^
	V1	L	4,304.52 (809.99)	4,029.64 (555.75)	0.168
		R	5,028.84 (910.81)	4,438.44 (748.37)	0.016^*^
	V2	L	11,443.04 (1411.91)	11,333.40 (179.85)	0.811
		R	11,739.20 (1508.17)	11,252.44 (1635.50)	0.279
Emotion/memory-related structures	Amygdala	L	1,792.36 (239.09)	1,628.16 (200.21)	0.011^*^
		R	1,808.84 (197.44)	1,800.84 (381.50)	0.926
	Hippocampus	L	4,412.52 (318.54)	4,356.44 (430.03)	0.603
		R	4,561.32 (350.98)	4,483.92 (327.82)	0.424

Volume in mm^3^, (Mean and S.D.). *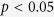.

**Table 4:** Relationship between abnormal brain morphological measures and clinical measures.

	Side		LGN_Vol_L	LGN_Vol_R	V1_Thick_R	Amygdala_Vol_L	FPC_Thick_R
**RNFL thickness**	L	***r***	0.221	0.522	0.267	−0.311	0.349
		***p***	0.515	0.099**	0.428	0.352	0.292
	R	***r***	0.047	0.502	−0.270	−0.312	−0.130
		***p***	0.892	0.116	0.421	0.350	0.704
**CDR**	L	***r***	−0.643	0.049	−0.403	0.183	0.489
		***p***	0.033*	0.885	0.220	0.590	0.127
	R	***r***	−0.610	−0.091	−0.670	−0.207	0.545
		***p***	0.046*	0.790	0.024*	0.542	0.083
**Visual field MD**	L	***r***	0.367	0.534	0.251	−0.457	−0.314
		***p***	0.267	0.091**	0.456	0.158	0.348
	R	***r***	0.104	0.699	−0.096	0.276	−0.029
		***p***	0.761	0.017*	0.780	0.412	0.933

LGN_Vol_L/R: left/right LGN volume, V1_Thick_R: right V1 cortical thickness, Amygdala_Vol_L: left amygdala volume. FPC_Thick_R: right FPC cortical thickness, RNFL: retinal nerve fiber layer, CDR: cup-to-disk ratio, MD: mean deviation. *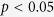, **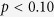.
